# Kinetic and temporospatial gait parameters in a heterogeneous group of dogs

**DOI:** 10.1186/s12917-015-0631-2

**Published:** 2016-01-04

**Authors:** Washington T. Kano, Sheila C. Rahal, Felipe S. Agostinho, Luciane R. Mesquita, Rogerio R. Santos, Frederico O. B. Monteiro, Maira S. Castilho, Alessandra Melchert

**Affiliations:** 1 Department of Veterinary Surgery and Anesthesiology, School of Veterinary Medicine and Animal Science – Univ Estadual Paulista (UNESP), Botucatu, SP Brazil; 2 Instituto de Saúde e Produção Animal, Universidade Federal Rural da Amazônia, Belém do Pará, Brazil; 3 Department of Veterinary Clinic, School of Veterinary Medicine and Animal Science – Univ Estadual Paulista (UNESP), Botucatu, SP Brazil

**Keywords:** Locomotion, Canine, Velocity, Objective measurement

## Abstract

**Background:**

A prime concern of the gait analysis in a heterogeneous group of dogs is the potential influence of factors such as individual body size, body mass, type of gait, and velocity. Thus, this study aimed to evaluate in a heterogeneous group of dogs a possible correlation of the stride frequency with kinetic and temporospatial variables, as well as the percentage of body weight distribution (%BWD), and compare symmetry index (SI) between trotting and walking dogs. Twenty-nine clinically healthy dogs moving in a controlled velocity were used. The dogs were organized into two groups based on duty factor. Group 1 comprised 15 walking dogs, aged from 9 months to 8 years and weighing about 22.3 kg. Group 2 had 14 trotting dogs, aged from 1 to 6 years and weighing about 6.5 kg. The kinetic data and temporospatial parameters were obtained using a pressure-sensing walkway. The velocity was 0.9–1.1 m/s. The peak vertical force (PVF), vertical impulse (VI), gait cycle time, stance time, swing time, stride length, and percentages of body weight distribution among the four limbs were determined. For each variable, the SIs were calculated. Pearson’s coefficient was used to evaluate correlation between stride frequency and other variables, initially in each group and after including all animals.

**Results:**

Except for the %BWD (approximately 60 % for the forelimbs and 40 % for the hind limbs), all other parameters differed between groups. Considering each Group individually a strong correlation was observed for most of the temporospatial parameters, but no significant correlation occurred between stride frequency and PVF, and stride frequency and %BWD. However, including all dogs a strong correlation was observed in all temporospatial parameters, and moderate correlation between stride frequency and VI, and weak correlation between stride frequency and PVF. There was no correlation between stride frequency and %BWD. Groups 1 and 2 did not differ statistically in SIs.

**Conclusions:**

In a heterogeneous group of dogs conducted at a controlled velocity, the %BWD and most of SIs presented low variability. However, %BWD seems to be the most accurate, since factors such as the magnitude of the variables may influence the SIs inducing wrong interpretation. Based on results obtained from correlations, the standardization of stride frequency could be an alternative to minimize the variability of temporospatial parameters.

## Background

A prime concern of the gait analysis using temporospatial parameters and kinetic data in a heterogeneous group of dogs is the potential influence of factors such as individual body size, body mass, type of gait, and velocity [1–5]. However, temporospatial parameters and kinetic data are important for identification and understanding of orthopedic problems, and for evaluating treatment response [6–8]. In addition, spatiotemporal characteristics have be used to evaluate gait in dogs with spinal cord disease, and may be useful as outcome measures for functional analysis in these patients [9, 10].

Given the relationship of limb length with the values of stance time, swing time, gait cycle time and stride length, the ratio between values can be changed by increasing stride frequency or the type of locomotion [5, 8]. This dynamic hampers the use of these parameters in comparisons due to the variability of the data. To walk at the same velocity as large dogs, small dogs require a higher stride frequency [3, 5]. Besides interfering directly in temporal values, such an increase in stride frequency may modify the ratio between stance time and swing time [1].

On the other hand, kinetic variables such as the PVF and VI may also be influenced by velocity and acceleration, body weight, animal conformation, and musculoskeletal structure [2, 6, 7, 11, 12]. One strategy to minimize the variability is to normalize the vertical force with canine body weight [1, 3, 5, 6, 12], but differences in individual size and, consequently, the relative velocity can still interfere with the values [3, 4]. However, a linear relationship may exist between kinetic variables and stride frequency that it is independent of the animal’s size and gait velocity [5].

In addition, calculus and normalization can be performed in order to minimize variations and provide parameters more apt for comparisons [1, 3, 13]. An index of symmetry or asymmetry can be used as an indicator of limb function while different evaluation methods have been employed in dogs [5, 8, 13–18]. In healthy animals it is expected that values of the variables obtained from the right and left forelimbs or between the right and left hind limbs are similar, consequently yielding a SI near 0, or perfect symmetry [8, 18].

Thus, the present study aimed to evaluate in a heterogeneous group of dogs a possible correlation of the stride frequency with kinetic and temporospatial variables, as well as the %BWD, and compare SI between trotting and walking dogs. The first hypothesis was that the stride frequency would have a linear correlation with the temporospatial parameters such as time and % of stance, time and % of swing, gait cycle time, and stride length. The second hypothesis was that the % BWD and SI would show a low variability in a heterogeneous group of dogs, and would not be affected by the stride frequency.

## Methods

### Dog selection

This study was approved by the Ethics Committee of School of Veterinary Medicine and Animal Science – Univ Estadual Paulista (UNESP) (no. 27/2014-CEUA). A signed Informed Consent Form was requested from each dog’s owner, prior to entering the study. Twenty-nine clinically healthy dogs moving in a controlled velocity were used. The dogs were organized into two groups based on duty factor. Group 1 comprised 15 walking dogs (duty factor >0.5), eight males and seven females, aged from 9 months to 8 years (mean ± SD, 3.3 y ± 2) and weighing about 22.3 kg (±10 SD). The dog breeds were Labrador retriever (*n* = 3), Pointer (*n* = 3), and eight crossbreeds. Group 2 had 14 trotting dogs (duty factor <0.5), six males and eight females, aged from 1 to 6 years (mean ± SD, 3.1 y ± 1.6) and weighing about 6.5 kg (±4.7 SD). The dog breeds were Shitsu (*n* = 3), Poodle (*n* = 2), Lhasa apso (*n* = 1), Dachshund (*n* = 1) and seven crossbreeds.

The dogs were judged to be healthy on account of results of complete physical and orthopedic examinations, and radiographic exams of the hip and elbow joints. Before data collection, the dogs were familiarized with the environment and pressure-sensing walkway, performing approximately five to seven practice trials. Each dog was weighed on the same electronic scale immediately before data collection.

### Data collection

The kinetic and temporospatial parameters of gait were measured on a 1.95 m x 0.45 m pressure-sensing walkway (Walkway High Resolution HRV4; Tekscan, South Boston, Massachusetts, USA), whose sensors were equilibrated and calibrated as specified by the manufacturer. Designated software (Walkway 7.0 software; Tekscan Inc., South Boston, Massachusetts, USA) was used for data acquisition and analysis.

The dogs were guided across the pressure-sensing walkway in a straight line on a loose leash to the left of the handler. For both groups, the velocity was maintained between 0.9 and 1.1 m/s, and the acceleration was between−0.2 and 0.2 m/s^2^. For each dog, an average of 20 trials was obtained, and first five valid trials were selected. A trial was considered valid if the four limbs had contacted the walkway surface during each gait cycle without the dog turning the head or pulling on the leash.

The temporospatial parameters evaluated for each limb were the gait cycle time (s), stance time (s) swing time (s) and stride length (m), as previously described [19]. The stance time percentage was determined from the following formula: (stance time/gait cycle time) x 100. The swing time percentage was calculated as follows: (swing time/gait cycle time) x 100. The stride corresponded to the distance between two consecutive ground contacts by the same limb. The duty factor was established by dividing stance time by gait cycle time. The stride frequency expressed in cycles per minute was defined as follows: (1/stance time) x 60.

The PVF and the VI were the kinetic parameters evaluated. The PVF and VI were normalized to the dog’s body weight and represented as a percentage of body weight. The % BWD among the four limbs was calculated as follows: (PVF of the limb/total PVF of the four limbs) x 100.

The SI between right and left side for both forelimbs and hind limbs for each kinetic and temporospatial variable was calculated as previously described [14] using the following equation:

$$ \mathrm{S}\mathrm{I}=\frac{1}{2}\left(\frac{\mathrm{RS}-\mathrm{L}\mathrm{S}}{\mathrm{RS}+\mathrm{L}\mathrm{S}}\right)\times 100 $$

RS: Right side
LS: Left side

The value of SI = 0 indicates perfect gait symmetry. Values of SI > 0 indicate asymmetry for the right limb, and values of SI < 0 indicate asymmetry for the left limb

### Statistical analysis

The normality of data was checked by the Shapiro-Wilk test. To compare the temporospatial parameters and the kinetic data between groups, the F-test was used followed by the Student’s *t* test. To evaluate the SIs between groups Mann–Whitney test was used. Differences were considered significant at *p* < 0.05.

Pearson’s correlation coefficients (r) were used to evaluate the linear relationships between the stride frequency and the other variables, initially in each group and after including all animals. The correlations were deemed significant at the 5 % probability level.

The kinetic and temporospatial values were expressed as the means ± standard deviation, and the inter-dogs coefficients of variation (CV) were calculated. The SIs were expressed as median, first quartile and third quartiles.

## Results

**Fig. 1:**
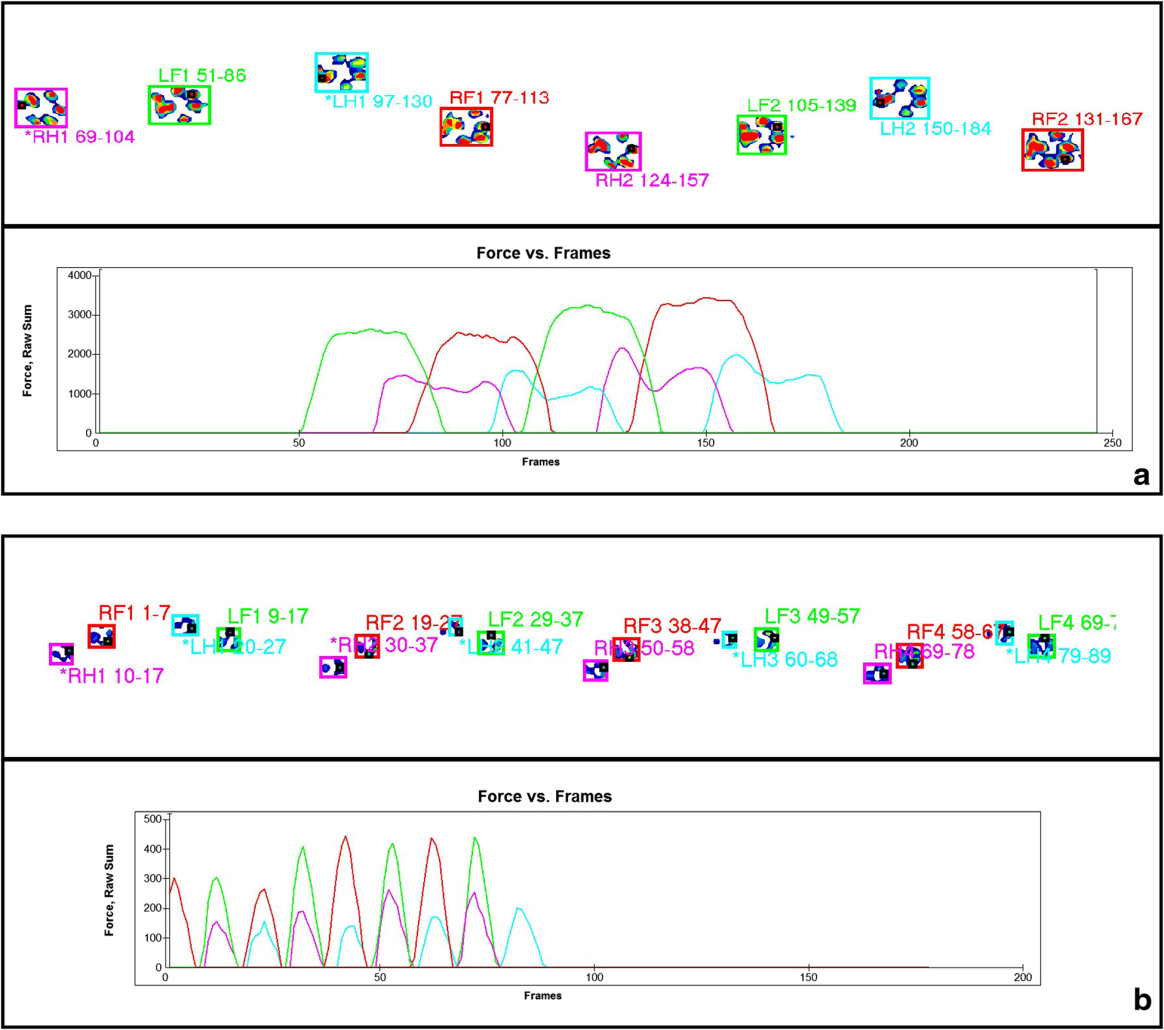
Representative recordings of a dog of Group 1 (**a**: walking) and Group 2 (**b**: trotting) on a pressure-sensing walkway

The dogs of Group 1 (walking) and Group 2 (trotting) showed significant differences in the kinetic and temporospatial parameters in both forelimbs (Table 1) and hind limbs (Table 2). However, no difference was observed for % BWD between groups. The mean %BWD including all dogs were 29.9 and 20.1 for forelimbs and hind limbs, respectively. Representative recordings of a dog of each Group on a pressure-sensing walkway is in Fig. 1.

**Table 1:** Comparison of the kinetic data and temporospatial parameters of the forelimbs between Groups 1 (walking) and 2 (trotting)

	Group 1		Group 2		
	Mean ± SD	CV	Mean ± SD	CV	*P value*
Stance Time (s)	0.46 ± 0.06	14.15	0.21 ± 0.06	27.47	<0.001
Swing Time (s)	0.28 ± 0.03	10.32	0.23 ± 0.04	18.12	<0.001
Gait cycle time (s)	0.73 ± 0.09	12.14	0.44 ± 0.09	20.90	<0.001
Stride Length (m)	0.74 ± 0.11	14.34	0.45 ± 0.10	21.88	<0.001
% of Stance	62.51 ± 2.55	4.08	46.86 ± 5.22	11.13	<0.001
% of Swing	38.58 ± 2.67	6.93	52.53 ± 5.42	10.32	<0.001
PVF (%BW)	74.45 ± 20.77	27.90	108.84 ± 35.99	33.06	<0.001
VI (%BW*s)	24.51 ± 8.54	34.85	15.00 ± 7.30	48.68	<0.001
% of Body Weight Distribution	30.00 ± 1.47	4.92	29.68 ± 2.28	7.68	0.505

*CV* coefficient of variation

**Table 2:** Comparison of the kinetic data and temporospatial parameters of the hind limbs between Groups 1 (walking) and 2 (trotting)

	Group 1		Group 2		
	Mean ± SD	CV	Mean ± SD	CV	*P value*
Stance Time (s)	0.45 ± 0.06	12.86	0.18 ± 0.05	26.89	<0.001
Swing Time (s)	0.30 ± 0.03	10.97	0.26 ± 0.04	15.60	<0.001
Gait cycle time (s)	0.75 ± 0.09	11.91	0.43 ± 0.08	18.91	<0.001
Stride Length (m)	0.73 ± 0.11	14.95	0.45 ± 0.11	24.23	<0.001
% of Stance	60.13 ± 2.05	3.41	40.46 ± 4.28	10.58	<0.001
% of Swing	40.65 ± 2.29	5.64	59.77 ± 4.53	7.59	<0.001
PVF (%BW)	50.67 ± 17.40	34.35	74.63 ± 26.12	35.00	<0.001
VI (%BW*s)	15.63 ± 6.21	39.71	8.26 ± 3.79	45.83	<0.001
% of Body Weight Distribution	20.00 ± 1.40	7.00	20.32 ± 2.24	11.01	0.508

*CV* coefficient of variation

The linear correlation values between stride frequency and kinetic and temporospatial variables for the dogs of Group 1 (walking), Group 2 (trotting) and including all dogs are described in Tables 3, 4 and 5, respectively. Considering each Group individually a strong correlation was observed for most of the temporospatial parameters, but no significant correlation occurred between stride frequency and PVF, and stride frequency and %BWD. However, including all dogs a strong correlation was observed in all temporospatial parameters, and moderate correlation between stride frequency and VI, and weak correlation between stride frequency and PVF. There was no correlation between stride frequency and % BWD.

**Table 3:** Pearson correlation coefficient and P value of the correlations between kinetic data or temporospatial parameters and stride frequency of the forelimbs and hind limbs in dogs of Group 1 (walking)

	Forelimb		Hind limb	
	*Coefficient*	*P value*	*Coefficient*	*P value*
Stance Time (s)	−0.974	<0.001	−0.947	<0.001
Swing Time (s)	−0.843	<0.001	−0.817	<0.001
Gait cycle time (s)	−0.991	<0.001	−0.991	<0.001
Stride Length (m)	−0.9	<0.001	−0.904	<0.001
% of Stance	−0.35	0.058	−0.259	0.167
% of Swing	0.484	0.007	0.443	0.014
PVF (%BW)	0.004	0.983	−0.048	0.803
VI (%BW*s)	−0.452	0.012	−0.424	0.012
% of Body Weight Distribution	0.041	0.831	−0.056	0.769

**Table 4:** Pearson correlation coefficient and *P* value of the correlations between kinetic data or temporospatial parameters and stride frequency of the forelimbs and hind limbs in dogs of Group 2 (trotting)

	Forelimb		Hind limb	
	*Coefficient*	*P value*	*Coefficient*	*P value*
Stance Time (s)	−0.91	<0.001	−0.903	<0.001
Swing Time (s)	−0.841	<0.001	−0.869	<0.001
Gait cycle time (s)	−0.97	<0.001	−0.952	<0.001
Stride Length (m)	−0.924	<0.001	−0.902	<0.001
% of Stance	−0.562	0.001	−0.688	<0.001
% of Swing	0.672	<0.001	−0.637	<0.001
PVF (%BW)	−0.104	0.583	0.131	0.49
VI (%BW*s)	−0.729	<0.001	−0.681	<0.001
% of Body Weight Distribution	−0.149	0.433	0.25	0.183

**Table 5:** Pearson correlation coefficient and *P* value of the correlations between kinetic data or temporospatial parameters and stride frequency of the forelimbs and hind limbs including both groups

	Forelimb		Hind limb	
	*Coefficient*	*P value*	*Coefficient*	*P value*
Stance Time (s)	−0.94	<0.001	−0.94	<0.001
Swing Time (s)	−0.84	<0.001	−0.81	<0.001
Gait cycle time (s)	−0.97	<0.001	−0.97	<0.001
Stride Length (m)	−0.94	<0.001	−0.93	<0.001
% of Stance	−0.83	<0.001	−0.87	<0.001
% of Swing	0.86	<0.001	0.87	<0.001
PVF (%BW)	0.36	0.004	0.44	0.001
VI (%BW*s)	−0.67	<0.001	−0.67	<0.001
% of Body Weight Distribution	−0.11	0.403	0.16	0.221

**Fig. 2:**
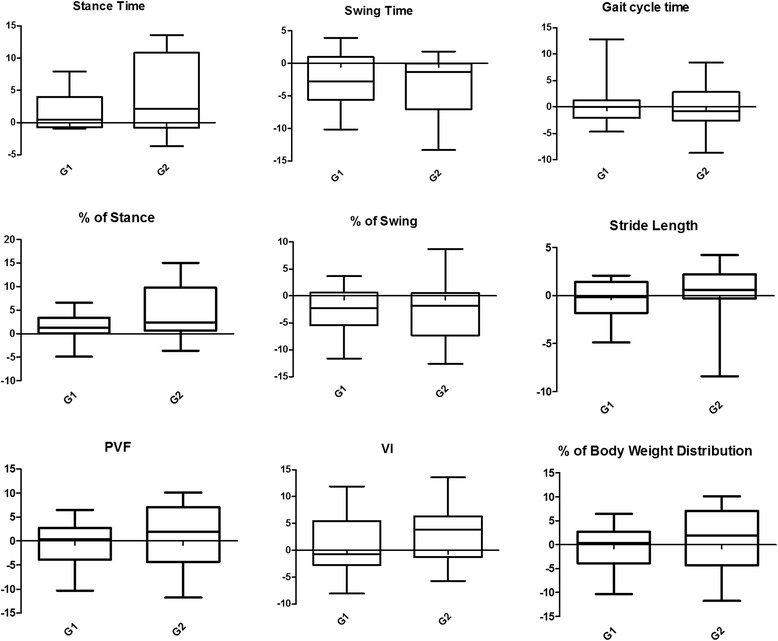
Boxplot of the kinetic data and temporospatial parameters of the forelimbs including both groups

**Fig. 3:**
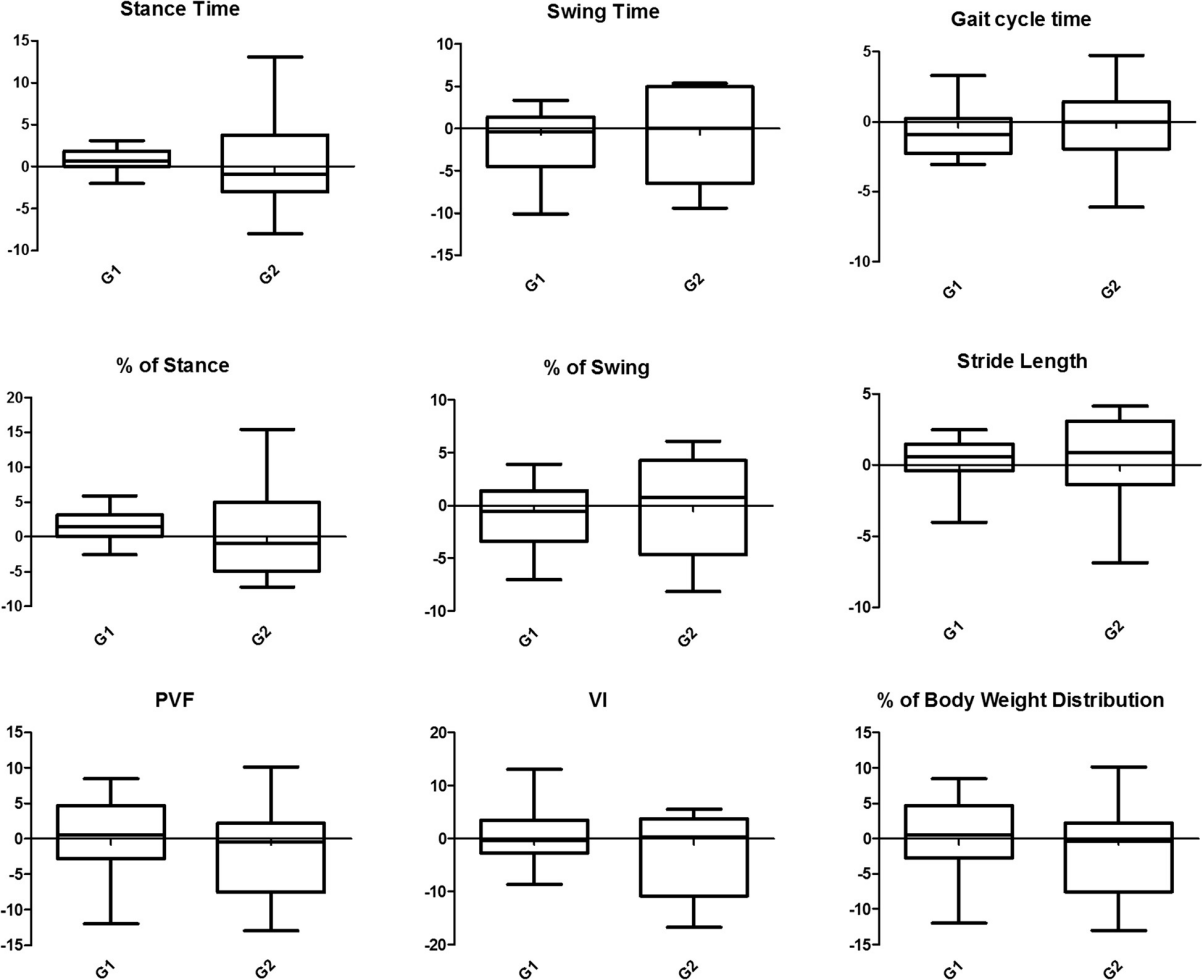
Boxplot of the kinetic data and temporospatial parameters of the hind limbs including both groups

Groups 1 and 2 did not differ statistically in SIs. For both groups 1 and 2, median, first quartile and third quartiles of SIs are described in Tables 6 and 7, respectively, for the forelimbs and hind limbs. Box plots with median, interquartile range, and maximum and minimum values are in Figs. 2 and 3, respectively, for the forelimbs and hind limbs.

**Table 6:** Comparison of the symmetry indices (%) of the kinetic data and temporospatial parameters of the forelimbs between Groups 1 (walking) and 2 (trotting)

	Group 1			Group 2			*P value*
	Median	Interquartile range		Median	Interquartile range		
		First Quartile	Third Quartile		First Quartile	Third Quartile	
Stance Time (s)	0.48	−0.63	3.52	2.11	0.00	10.81	0.369
Swing Time (s)	−2.73	−5.17	0.5	−1.32	−4.65	0.00	0.884
Gait cycle time (s)	−0.01	−1.83	1.05	−0.83	−1.65	2.58	0.923
Stride Length (m)	−0.16	−1.42	1.18	0.59	−0.09	2.12	0.190
% of Stance	1.26	−0.19	3.18	2.41	1.04	9.39	0.190
% of Swing	−2.29	−4.99	0.31	−1.85	−6.71	−0.03	0.698
PVF (%BW)	0.35	−3.24	2.46	1.98	−2.61	5.79	0.438
VI (%BW*s)	−0.67	−2.66	3.78	3.83	−0.22	6.11	0.382
% of Body Weight Distribution	0.35	−3.24	2.46	1.98	−2.61	5.79	0.438

**Table 7:** Comparison of the symmetry indices (%) of the kinetic data and temporospatial parameters of the hind limbs between Groups 1 (walking) and 2 (trotting)

	Group 1			Group 2			*P value*
	Median	Interquartile range		Median	Interquartile range		
		First Quartile	Third Quartile		First Quartile	Third Quartile	
Stance Time (s)	0.68	0.00	1.69	−0.93	−2.41	3.56	0.771
Swing Time (s)	−0.35	−4.36	1.12	0.00	−4.76	4.91	0.466
Gait cycle time (s)	−0.88	−2.01	0.24	0.00	−1.46	1.34	0.645
Stride Length (m)	0.60	−0.32	1.45	0.90	−1.18	3.01	0.357
% of Stance	1.47	0.16	2.85	−0.93	−4.67	4.39	0.627
% of Swing	−0.50	−3,26	0.98	0.77	−4.55	4.02	0.409
PVF (%BW)	0.52	−2.47	3.84	−0.41	−4.96	2.05	0.409
VI (%BW*s)	−0.41	−2.19	3.30	0.36	−10.46	2.76	0.357
% of Body Weight Distribution	0.52	−2.47	3.84	−0.41	−4.96	2.05	0.409

## Discussion

Several variables must be controlled to avoid variability in kinetic data and temporospatial parameters, including velocity and type of locomotion [1, 17, 20, 21], stance time [21], training and habituation [22], body size, conformation, and body weight [1, 2, 4, 5]. In addition, most of the kinetic studies have evaluated dogs that were walking or trotting, due to the symmetry and convenience of these types of locomotion [5, 7, 16, 17]. In the present study, the velocity was maintained 0.9–1.1 m/s and the acceleration between−0.2 and 0.2 m/s^2^ determined by pressure-sensitive walkway. A training program was not performed in the present study. Because the data are more easily collected using a pressure-sensing walkway compared to a single force plate, the measurements are generally obtained after familiarization to pressure-sensing walkway than a training program [5, 16].

The center of gravity in dogs is located next to the forelimbs possibly near the base of the heart, so that in a healthy dog 60 % of the weight is carried by the forelimbs [23]. The body weight distributions reported in a study of healthy dogs walking on a pressure-sensing walkway, were 60.7 and 39.3 % for small dogs and 61.7 and 38.3 % for large dogs, respectively, for the forelimbs and hind limbs, without influence of body weight or size [5]. In the present study, the mean body weight distributions were similar, being 30 % (G1) and 29.7 % (G2) for each forelimb, and 20 % (G1) and 20.3 % (G2) for each hind limb. Thus, the %BWD may be applicable to comparisons in a heterogeneous group of dogs, because regardless of the body weight, body size, and gait types the values are maintained.

Since the velocity was controlled in the present study, the stride frequency was used to calculate the Pearson correlation coefficients. Besides, the stride frequency is an objective variable calculated by the system, and errors that may occur with tape measurements of the limbs are avoided. The Pearson correlation revealed a strong correlation in all temporospatial parameters analyzing all dogs as unique group, more than analyzing each group individually; suggesting that the gait type did not interfered in this correlation.

The Pearson correlation revealed a strong negative correlation between stride frequency and most temporospatial parameters. Therefore, the values of stance time, swing time, gait cycle time, stride length decreased as stride frequency increased. A study comparing small and large dogs walking at their preferred velocity on a pressure-sensing walkway also reported that most of the temporospatial parameters (gait cycle time, stance time and swing time) were lower for small dogs [5]. On the other hand, a strong positive correlation with swing percentage and a highly negative correlation with stance time percentage were found. Thus, as stride frequency increases, the limb spends proportionately less time on the ground and more time off the ground. Conversely, it was reported that in quadrupeds the swing phase diminishes with increased velocity whereas during trotting and galloping the parameter is quite constant [24].

With respect to the kinetic parameters, the PVF and VI showed, respectively, low correlation and moderate coefficient values indicating a weaker relationship with stride frequency. A previous study using healthy dogs found that PVF was elevated as the velocity increased and decreased as the stance time increased, while VI decreased as the velocity increased and increased as stance time increased [21]. Thus, other factors may influence PVF and VI, and these parameters may not useful in a heterogeneous group of dogs. On the other hand, no significant correlation was observed between stride frequency and the %BWD, suggesting that the latter parameter was not influenced by the stride frequency.

Symmetry or asymmetry indices or symmetry rates have been used to evaluate kinetic data and temporospatial parameters in dogs walking or trotting over a pressure-sensing walkway, aiming to characterize healthy dogs of the same size or different sizes [5, 17], or to distinguish between lame dogs and clinically healthy dogs [16, 18]. This same strategy was employed in the present study in order to assess the validity of SI in heterogeneous group of dogs, but under controlled velocity.

In both groups the SI of all variables showed median values nearly 0 and asymmetry less than 4 % showing no differences between Groups 1 and 2. These data suggest that these indices could be utilized to evaluate the gait in a heterogeneous group of dogs. However, some facts can limit the use SI for comparison between groups.

A major problem with the SI is that precision depends on the relative magnitude of the evaluated variable [14]. If the magnitude of the variable itself is quite small, such as temporal gait variables in trotting dogs, even small differences may result in high value of SI. Probably, these differences are clinically insignificant, or may be resultant of capture artefacts. On the other, SI of the gait cycle time could be used as an indicative of capture artefacts, since at a constant velocity is not expected asymmetry in this variable.

As an example, the gait cycle time of the forelimb in Group 2 showed 2.58 % of asymmetry (third quartile), which represented a difference of approximately 0.04 s of the mean value of this variable (0.44 s). This difference in mean value of stance phase (0.21 s) can result in a SI of 6 %, and if applied in the dog that showed the lower stance phase (0.13 s) the SI will be 9.1 %. This could explain the high variation of temporal variable SI as well as the SI of VI (total force applied overtime) in Group 2.

In addition, PVF and %BW showed equal values of SIs with a median value near 0. However, a high maximum values can be observed in the boxplots, especially in Group 2. The magnitude of the variable could be a reason to the higher variation in Group 2, but other factors such as velocity variations not evident in trials [14] and no previous training [22] must be considered.

## Conclusions

In a heterogeneous group of dogs conducted in a controlled velocity, the %BWD and most of SIs presented low variability. However, %BWD seems to be the most accurate, since factors such as the magnitude of the variables may influence the SIs inducing wrong interpretation. Based on results obtained from correlations, the standardization of stride frequency could be an alternative to minimize the variability of temporospatial parameters. The identification of a linear correlation between stride frequency and other variables may be an option for future studies aiming a determination of a correction factor.

Therefore, of all of the studied variables the %BDW is the most useful and accurate for clinicians to evaluate a heterogeneous group of dogs since this variable is not influenced by stride frequency.

## Abbreviations

%BWD: Percentage of body weight distribution
PVF: Peak vertical force
VI: Vertical impulse
SI: Symmetry index
SIs: Symmetry indices
